# Convergent evolution of SARS-CoV-2 in human and animals

**DOI:** 10.1007/s13238-021-00847-6

**Published:** 2021-04-30

**Authors:** Hang-Yu Zhou, Cheng-Yang Ji, Hang Fan, Na Han, Xiao-Feng Li, Aiping Wu, Cheng-Feng Qin

**Affiliations:** 1 Center for Systems Medicine, Institute of Basic Medical Sciences, Chinese Academy of Medical Sciences & Peking Union Medical College, Beijing, 100005 China; 2 Suzhou Institute of Systems Medicine, Suzhou, 215123 China; 3 State Key Laboratory of Pathogen and Biosecurity, Beijing Institute of Microbiology and Epidemiology, AMMS, Beijing, 100071 China

Recently, many SARS-CoV-2 variants including 501Y.V1, 501Y.V2 and 501Y.V3 were detected in different regions (Table S1) and drew great attention from all over the world. The 501Y.V1 was firstly isolated in the United Kingdom (UK) (Davies et al., 2020) and featured with 7 substitutions including N501Y as well as 3 deletions in S protein. This variant was identified to increase the viral transmissibility by 56% in comparison with the preexisting strains. Days after this report, another SARS-CoV-2 variant (501Y.V2) featured with N501Y, K417N and E484K substitutions in S protein was supposed to rapidly outcompete the preexisting strains (Tegally et al., 2020) in South Africa. Besides, the 501Y.V3 variant was initially detected in Brazil and has caused rapidly increased infections with SNPs N501Y, K417T and E484K. Of them, N501Y, K417N/T and E484K are of particular interest because the N501Y was shared in all three variants and the K417N/T and E484K were detected simultaneous appeared with N501Y in 501Y.V2 and 501Y.V3.

A coincidence is that the remarkable N501Y mutation also emerged during mouse adaption of SARS-CoV-2. Starting from a clinical isolation from COVID-19 patient, serial passaging of the virus in the lung of aged mice led to the emergence of a panel of adaptive mutations including N501Y (Gu et al., 2020). Deep sequencing showed that the N501Y mutation has readily been emerged after a single passage in mouse lung, and the proportion of mutation gradually increased during several subsequent passages. Structural modelling suggested the N501Y probably increased the binding affinity to mouse ACE2, which account for the increased mouse infectivity and virulence (Gu et al., 2020). With the help of deep mutation, Starrs et al. found N501Y mutation may also enhance the human ACE2 affinity of SARS-CoV-2 (Starr et al., 2020).

In the mice adaption model, the K417N could be observed to appear accompany with N501Y after serial passaged the native virus for 30 generations (Fig. 1A). This viral strain with both N501Y and K417N substitutions rapidly become the dominant strain in viral population (Fig. 1A) (Sun et al., 2020). Notably, this is also in accordance with the appearing order of these two mutations in humans. Viruses with N501Y substitutions has been identified in Brazil at Apr. 7th 2020, in the USA at Apr. 21st, and in the UK at Jun. 8th, while viruses with both N501Y and K417N were initially detected in the early of Oct. 2020. The rapid outcompete of viral strains with both N501Y and K417N compared with virus only with N501Y may provide reasonable explanation to the present outbreak of viral strains with both these two substitutions.

**Figure 1:**
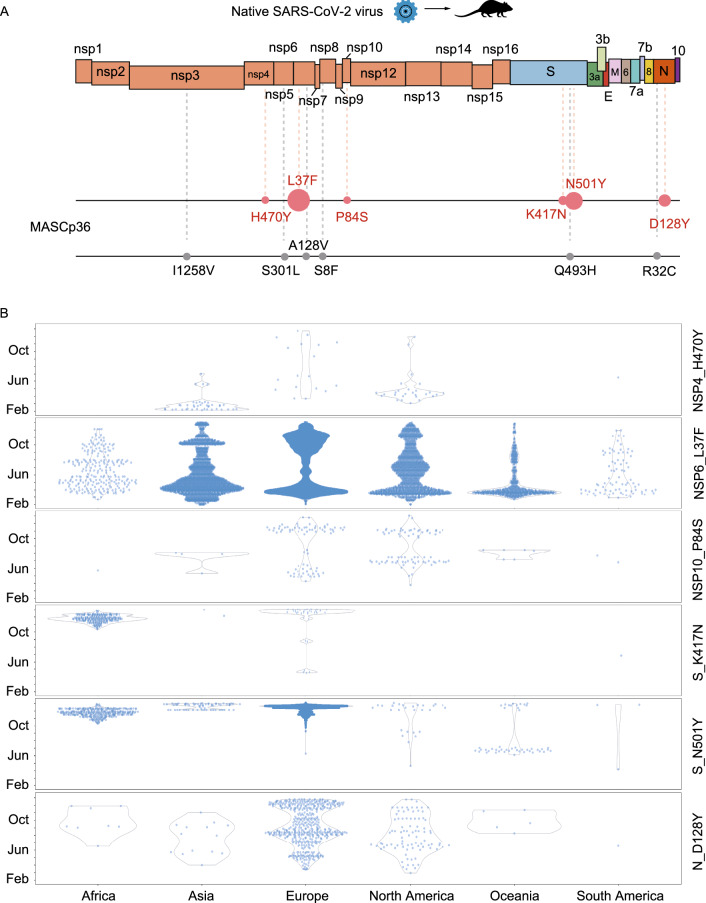
Shared mutations of SARS-CoV-2 strains in mouse and human. (A) After passaging a patient isolated virus in mice for 36 passages, 12 mutations could be detected. Six mutations detected both in mice and circulated in humans were colored red while other mutations could only be detected in mice were colored black. For the red dots, the size of dots was related with the frequency of each mutations in humans. (B) The spatio-temporal distribution in humans of the viral strains with the six mutations detected both in mice and human collected in GISAID until Jan. 11th 2021. H470Y could be detected in patients in Asia, Europe, North America and South America. L37F could be detected in viruses all over the world. P84S could be identified mainly in Europe and North America. The K417N mutation could be detected earlier in Europe but not detected continuously, after Oct. this mutation was bursting with N501Y in Africa. The bursting of N501Y mutation in Africa and Europe since Sep. related with the observed pandemic caused by 501Y.V1 and 501Y.V2. The D128Y were mainly occurred in Europe and North America

Besides the two focused substitutions in S protein, four other amino acid substitutions were found appeared both in the mouse adapted virus strain (MASCp36) and in humans (data updated till Jan. 11th 2021), including H470Y in nsp4, L37F in nsp6, P84S in nsp10 and D128Y in N protein of SARS-CoV-2. The spatio-temporal distribution of viral strains with the six mutations collected in GISAID until Jan. 11th 2021 were analyzed (Elbe and Buckland‐Merrett, 2017; Shu and McCauley, 2017). It should be noted that the L37F was detected repeatedly in virus all over the world, while the P84S and D128Y always existed in humans (Fig. 1B). Although the biological impact of these mutations remain elusive, the coincidental appearance of the same pattern of mutations detected in mice adaption model and COVID-19 patients reminds us the potentially indicative value of mouse adaption model in monitoring viral circulation and adaption in humans.

Meanwhile, another mouse adaption study conducted by Baric and colleagues (Leist et al., 2020) also detected a panel of adaptive mutations in mouse model. In total, 4 mutations including T285I in nsp4, K2R in nsp7, E23G in nsp8 and F7S in ORF6 were identified after 10 *in-vivo* passage. Of the 4 detected mutations, T285I was observed to be appeared in COVID-19 patients, whereas other 3 mutations seem to be only observed in mice. Strikingly, the N501Y mutation was not recorded in this study. The divergence between two passaging studies was probably associated with the starting virus. Virus used in Baric’s study was not a wild-type viral strain, but an engineered SARS-CoV-2 strain with Q493K mutation in S protein. Previously, mouse adapted viruses were also reported in SARS-CoV and MERS-CoV mouse models (Cockrell et al., 2016; Roberts et al., 2007). No convergent evolution was detected in human and mouse model of MERS-CoV, which might due to the engineered mouse in this experiment. However, in the mouse model of SARS-CoV that used a clinical SARS-CoV for serial passaging in mice, the only mutation in S protein of MA15 (Y436H) could also be detected in early SARS-CoV strain in human (Chinese SARS Molecular Epidemiology Consortium, 2004).

Additionally, recent findings from mink also provided clues for SARS-CoV-2 adaption. Munnink et al. reported the circulation and evolution of SARS-CoV-2 introduced from human within mink farm (Munnink et al., 2020). In their research, viral circulation in NB1 and NB2 farms were inferred to appear independently. After analyzing the mutations in minks from farms NB1 and NB2, 10 out of 20 mutations were detected to circulate in humans (Fig. S1). Notably, 4 out of 5 detected substitutions in S protein were also observed in viral strains isolated from human. The V367F detected in NB1 was identified to enhance S protein expression by Starrs et al (Starr et al., 2020), while the Y453F in S protein was occurred in mink-infecting strains and also appeared in more than 1,000 human-infecting SARS-CoV-2 strains.

One question is whether animal models can be used to study the original source of virus mutation? In some specific situations like the ferret farms in Netherlands (Munnink et al., 2020) with relative isolated environments, the mutation Y453F in S protein could be confirmed to be first mutated in animals and then transmitted to humans. However, In most cases, without detailed epidemiological information, animal models cannot be used to explore the original source of virus mutation.

Overall, these convergent mutations emerged during human transmission and animal adaption help to understand the key factors that have driven the rapid evolution of SARS-CoV-2. Traditionally, animal models are useful tools to evaluate viral virulence, pathogenic mechanism and vaccine effectiveness. However, recent animal adaption experiments indicate that mutations observed in isolated adaptive SARS-CoV-2 from infected animals may also provide valuable information for how virus adapts in the transmission of human society. Especially, those mutations in the S protein of native virus adaption may have the convergent substitutions among different hosts, such as mouse, mink or human. Since over 20,000 mutations of SARS-CoV-2 have been identified now and most of them may not have advantage of fitness in viral evolution (Grubaugh et al., 2020), the adapted mutations from animal models might help to filter the ”noise” in such a large amount of mutation pool. Learning these warning signs from animals might help respond quickly and prepare well for the emerging viral variants before their widespread transmission in humans. However, it also should be noted that viruses may undergo different mutations in order to adapt to different hosts. To what extent the adapted mutations in animal models to mimic those in humans requires further investigation.

## Supplementary Information

Below is the link to the electronic supplementary material.

Supplementary material 1 (PDF 235 kb)
