# Supplementary material for: Convergent evolution of SARS-CoV-2 in human and animals
Source: Protein Cell. 2021 Apr 30;12(11):832–5. doi: 10.1007/s13238-021-00847-6 (PMC8085470; doi:10.1007/s13238-021-00847-6)
Supplement: 13238_2021_847_MOESM1_ESM — Supplementary data [file 13238_2021_847_moesm1_esm.pdf]

Supplementary data

Table S1 Detailed information of recent SARS-CoV-2 variants.

PANGO lineages	Nextstrain clade	Other names	Featured mutations	Distribution	Sequences in GISAID*
B.1.1.7	20I/501Y.V1	VOC-202012/01	NSP3:T183I,A890D,I1412T<br>NSP6: Δ106-108<br>NSP12:P323L<br>S:N501Y,A570D,D614G,P681H,T716I,S982A,D1118H,Δ69-70,Δ144/145<br>ORF8:Q27stop,R52I,Y73C<br>N:D3L,R203K,G204R,S253F	Africa, Asia, Europe, North America, South America	161760
B.1.351	20H/501Y.V2	VOC-202012/02	NSP2:T85I<br>NSP3:K837N<br>NSP5:K90R<br>NSP6:Δ106-108<br>NSP12:P323L<br>S:D80A,D215G,K417N,E484K,N501Y,D614G,A701V,Δ242-244<br>ORF3a:Q57H,S171L<br>E:P71L<br>N:T205I	Africa, Asia, Europe, North America, South America	3885
P.1	20J/501Y.V3	VOC-202101/02	NSP3:S370L,K977Q<br>NSP6: Δ106-108<br>NSP12:P323L<br>NSP13:E341D<br>S:L18F,T20N,P26S,D138Y,R190S,K417T,E484K,N501Y,D614G,H655Y,T1027I,V1176F<br>ORF3a:S253P<br>ORF8:E92K<br>N:P80R,R203K,G204R	Asia, Europe, North America, South America	760
B.1.1.207	-	-	NSP6:A2V<br>NSP12:P323L<br>S:E484K,D614G,P681H<br>M:V70F<br>ORF3a:A143S<br>N:R203K,G204R	Asia, Europe, North America, South America	638
B.1.429	20C	CAL.20C	NSP2:T85I<br>NSP9:I65V<br>NSP12:P323L<br>NSP13:D260Y<br>S:W152C,L452R<br>ORF3a:Q57H<br>N:T205I	Asia, Europe, North America, South America	8600
B.1.525	-	VUI-202102/03	NSP3:T1189I<br>NSP6:Δ106-108<br>NSP12:P323F<br>S:Q52R,A67V,E484K,D614G,Q677H,F888L,Δ69-70,Δ144/145<br>E:L21F<br>M:I82T<br>N:A12G,T205I	Africa, Asia, Europe, North America	558
-	-	COH.20G/677H	NSP2:T85I<br>NSP3:M1788I<br>NSP5:L89F<br>NSP12:P323L<br>NSP14:N129D<br>NSP16:R216C<br>S:Q677H,D614G<br>ORF3a:Q57H,G172V<br>M:A85S<br>ORF8:S24L<br>N:P67S,P199L,D377Y	North America	722
-	-	COH.20G/501Y	NSP2:T85I<br>NSP3:T181I,A256V<br>NSP5:L89F<br>NSP12:P323L<br>NSP14:N129D<br>NSP16:R216C<br>S:N501Y,D614G<br>ORF3a:Q57H,G172V<br>ORF8:S24L,R52I<br>N:P67S,P199L	North America	5
-	-	Mink Cluster 5	NSP1:Δ85<br>NSP3: Δ1263<br>NSP12:P323L,T739I<br>NSP15:T112I<br>S:Y453F,I692V,M1229I,Δ69-70<br>ORF3a:H182Y<br>N:S194L,R203K,G204R	Europe	11

*The data updated until 17th Mar. 2021, mutations identified in different variants were colored red.

Figure S1

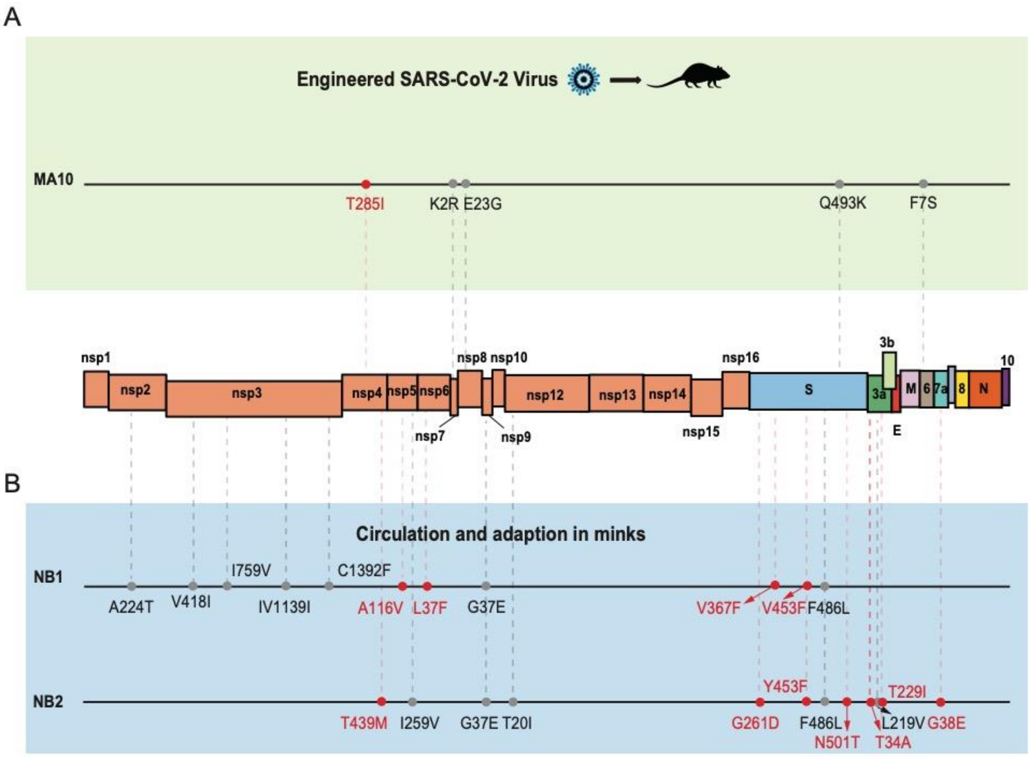

Figure S1 Mutations of SARS-CoV-2 strains during their adaption in mice and minks. (A) In the research conducted by Baric and colleagues, after 10 serial passage of an engineered SARS-CoV-2 virus (with Q493H mutation in S protein) in mice for 10 passages, 4 mutations including T285I in nsp4, K2R in nsp7, E23G in nsp8 and F7S in orf6 could be detected. Of the 4 mutations, the T285I mutation could be detected in human as well. (B) In the research conducted by Munnink et al., 20 mutations could be detected in minks isolated from NB1 and NB2 farms, in which 10 of them could also be found in human-infecting strains. The V453F could be detected both in NB1 and NB2 farms and could also be found in 916 viral strains circulated in human. Besides, T439M in nsp4, A116V in nsp5, L37F in nsp6, G261D, V367F, N501T in S, T34A, T229I in orf3a and G38E in orf7a were detected in human population as well.
